# Comparative Efficacy and Safety of Neuroprotective Therapies for Neonates With Hypoxic Ischemic Encephalopathy: A Network Meta-Analysis

**DOI:** 10.3389/fphar.2019.01221

**Published:** 2019-10-25

**Authors:** Clare Yuen Zen Lee, Pairote Chakranon, Shaun Wen Huey Lee

**Affiliations:** ^1^School of Pharmacy, Monash University Malaysia, Bandar Sunway, Malaysia; ^2^Faculty of Pharmacy, Silapakorn University, Pathom, Thailand; ^3^Asian Centre for Evidence Synthesis in Population, Implementation and Clinical Outcomes (PICO), Health and Well-being Cluster, Global Asia in the 21st Century (GA21) Platform, Monash University Malaysia, Selangor, Malaysia; ^4^School of Pharmacy, Taylor’s University, Subang Jaya, Malaysia

**Keywords:** hypoxic ischemic encephalopathy, neonatal, systematic review, meta-analysis, neuroprotective, perinatal

## Abstract

**Context:** Several interventions are available for the management of hypoxic ischemic encephalopathy (HIE), but no studies have compared their relative efficacy in a single analysis. This study aims to compare and determine the effectiveness of available interventions for HIE using direct and indirect data.

**Methods:** Large randomized trials were identified from PubMed, EMBASE, CINAHL Plus, AMED, and Cochrane Library of Clinical Trials database from inception until June 30, 2018. Two independent reviewers extracted study data and performed quality assessment. Direct and network meta-analysis of randomized controlled trials was performed to obtained pooled results comparing the effectiveness of different therapies used in HIE on mortality, neurodevelopmental delay at 18 months, as well as adverse events. Their probability of having the highest efficacy and safety was estimated and ranked. The certainty of evidence for the primary outcomes of mortality and mortality or neurodevelopmental delay at 18 months was evaluated using GRADE criteria.

**Results:** Fifteen studies comparing five interventions were included in the network meta-analysis. Whole body cooling [Odds ratio: 0.62 (95% credible interval: 0.46–0.83); 8 trials, high certainty of evidence] was the most effective treatment in reducing the risk of mortality, followed by selective head cooling (0.73; 0.48–1.11; 2 trials, moderate certainty of evidence) and use of magnesium sulfate (0.79; 0.20–3.06; 2 trials, low certainty of evidence). Whole body hypothermia (0.48; 0.33–0.71; 5 trials), selective head hypothermia (0.54; 0.32–0.89; 2 trials), and erythropoietin (0.36; 0.19–0.66; 2 trials) were more effective for reducing the risk of mortality and neurodevelopmental delay at 18 months (moderate to high certainty). Among neonates treated for HIE, the use of erythropoietin (0.36; 0.18–0.74, 2 trials) and whole body hypothermia (0.61; 0.45–0.83; 7 trials) were associated with lower rates of cerebral palsy. Similarly, there were lower rates of seizures among neonates treated with erythropoietin (0.35; 0.13–0.94; 1 trial) and whole body hypothermia (0.64; 0.46–0.87, 7 trials).

**Conclusion:** The findings support current guidelines using therapeutic hypothermia in neonates with HIE. However, more trials are needed to determine the role of adjuvant therapy to hypothermia in reducing the risk of mortality and/or neurodevelopmental delay.

## Introduction

Neonatal hypoxic ischemic encephalopathy (HIE) is one of the most common causes of severe neurological deficit in children, affecting an estimated 15 per 10,000 live births (Graham et al., 2008; American College of Obstetricians and Gynecologists, and American Academy of Pediatrics, 2014). Several reviews have suggested that therapeutic hypothermia (both whole body and selective head) reduces mortality and improves survival with normal neurological outcome and is now a standard treatment protocol for most neonatal centers in developed countries (Edwards et al., 2010; Jacobs et al., 2013; Martinello et al., 2017). Despite this, their effectiveness is still limited, with mortality rates of approximately 10% −20% in several large trials (Jacobs et al., 2013). As a result, alternative strategies, including the use of adjuvant therapies such as xenon, allopurinol, erythropoietin, magnesium sulfate, and melatonin, have been suggested (Perrone et al., 2012). Other strategies suggested include cooling for longer periods of time, cooling at lower temperature or both (Shankaran et al., 2017). 

Recently, several randomized controlled trials have examined the effects of such adjuvant therapy in neonates with HIE, yielding a complex evidence base that requires careful examination across different strategies (Bhat et al., 2009; Aly et al., 2015; Azzopardi et al., 2016; Filippi et al., 2017). However, most of these studies are relatively small and the results remains inconclusive and mixed. There are no reviews that attempted to summarize these data from large studies. 

Furthermore, previous meta-analyses have often compared the efficacy of treatment within pairs of active treatment, which provides limited insights into the overall treatment hierarchy as treatment effects are estimated from two different treatment comparison only (Jacobs et al., 2013; Pauliah et al., 2013). Over the past few years, the use of network meta-analyses which allows for the simultaneous comparison of two or more interventions has increasingly been used (Lee et al., 2015; Lee et al., 2017; Bukhsh et al., 2018; Teoh et al., 2019). Network meta-analysis includes both direct and indirect comparison within a single analysis, thereby providing an integrated and more holistic conclusion, providing decision makers with a more complete evidence matrix (Watt et al., 2019). In this study, we aimed to estimate the efficacy and safety of available neuroprotective interventions for HIE who participated in randomized controlled studies. 

## Materials and Methods

### Search Strategy

A literature search was performed to identify for studies from inception to June 30, 2018, on the following databases: PubMed, EMBASE, CINAHL Plus, Allied and Complementary Medicines (AMED), and the Cochrane Central Register of Controlled Trials without any language restriction. We also obtained additional records by reviewing the reference list of the retrieved articles and other resources including Google Scholar, NDLTD database, and ClinicalTrials.gov. A full list of search terms can be found in **eText** in **Supplementary Material**.

### Study Selection

Studies were considered eligible for inclusion if they (1) were randomized controlled studies (RCT), (2) recruited term or preterm infants (gestational age ≥35 weeks) diagnosed with HIE (3) had a control group or comparison group, (4) sufficiently powered to detect differences in the outcome of death and/or disability, and (5) the infants were given any of the following as intervention: magnesium sulfate, deferoxamine, cannabinoids, melatonin, statin, topiramate, xenon, allopurinol, erythropoietin, N-acetylcysteine, or therapeutic hypothermia; either as single intervention or adjunct therapy. Studies which had significant methodological limitations such as poor description of inclusion/exclusion criteria were excluded. 

### Data Collection and Extraction

All identified records were screened independently by titles and abstracts by two reviewers (CL and PC) and validated by another reviewer (SL). The full texts of relevant articles were retrieved for further eligibility assessment, extracted, and any discrepancies were resolved through discussion. The information extracted included the author, study design and population, outcomes, and quality of the study using a standardized data extraction form. We subsequently assessed the study quality using the Cochrane risk of bias assessment tool (Higgins et al., 2011). 

### Outcome Measures

The co-primary outcomes of interest were the composite of mortality or major neurodevelopmental disability and/or mortality assessed at least 18 months of age. Secondary outcomes include cerebral palsy, development delay based upon the mental development and psychomotor indices of the Bayley II scales of infant development (Bayley, 1993), seizures, quality of vision and hearing, and potential adverse effects caused by the treatment. 

### Statistical Analyses

We used a stepwise approach whereby traditional meta-analysis was performed using the Mantel-Haenszel random-effects model since we expect the presence of heterogeneity. We calculated the risk ratio and risk difference for dichotomous outcomes, and its 95% confidence interval. To determine whether the benefit of treatment on outcomes was affected by the severity of encephalopathy, we examined subgroups for which the severity of encephalopathy was graded as moderate or severe on the basis of clinical examination and/or amplitude integrated electroencephalography. The consistency of the treatment effect across subgroups was explored by calculating the ratio of relative risks with 95% confidence interval. Potential small study publication bias was assessed using visual inspection of the funnel plot and Eggers test. Between studies heterogeneity was assessed using *I* ^2^ and Cochran’s Q method. 

We subsequently performed a network meta-analysis which combines the direct and indirect effects of treatment, allowing for simultaneous comparison of multiple treatments. These were ranked using the surface under the curve ranking (SUCRA). Inconsistency checks were performed for closed loop in the network (Higgins et al., 2012; Veroniki et al., 2013). Subsequently, we calculated the number needed to treat (NNT) or number needed to harm (NNH) to better understand the potential benefits of different treatments examined. We used the odds ratios (OR) derived from the usual care comparison in network meta-analysis for mortality outcome to estimate the absolute benefits (Dulai et al., 2016). 

Preplanned sensitivity analyses include comparison between high versus low-middle income countries as well as severity of encephalopathy. All analyses were conducted using Stata version 13.0 (StataCorp, College TX). This study is registered with PROSPERO, number CRD42016053390.

## Results

### Study Selection and Characteristics

**Figure 1:**
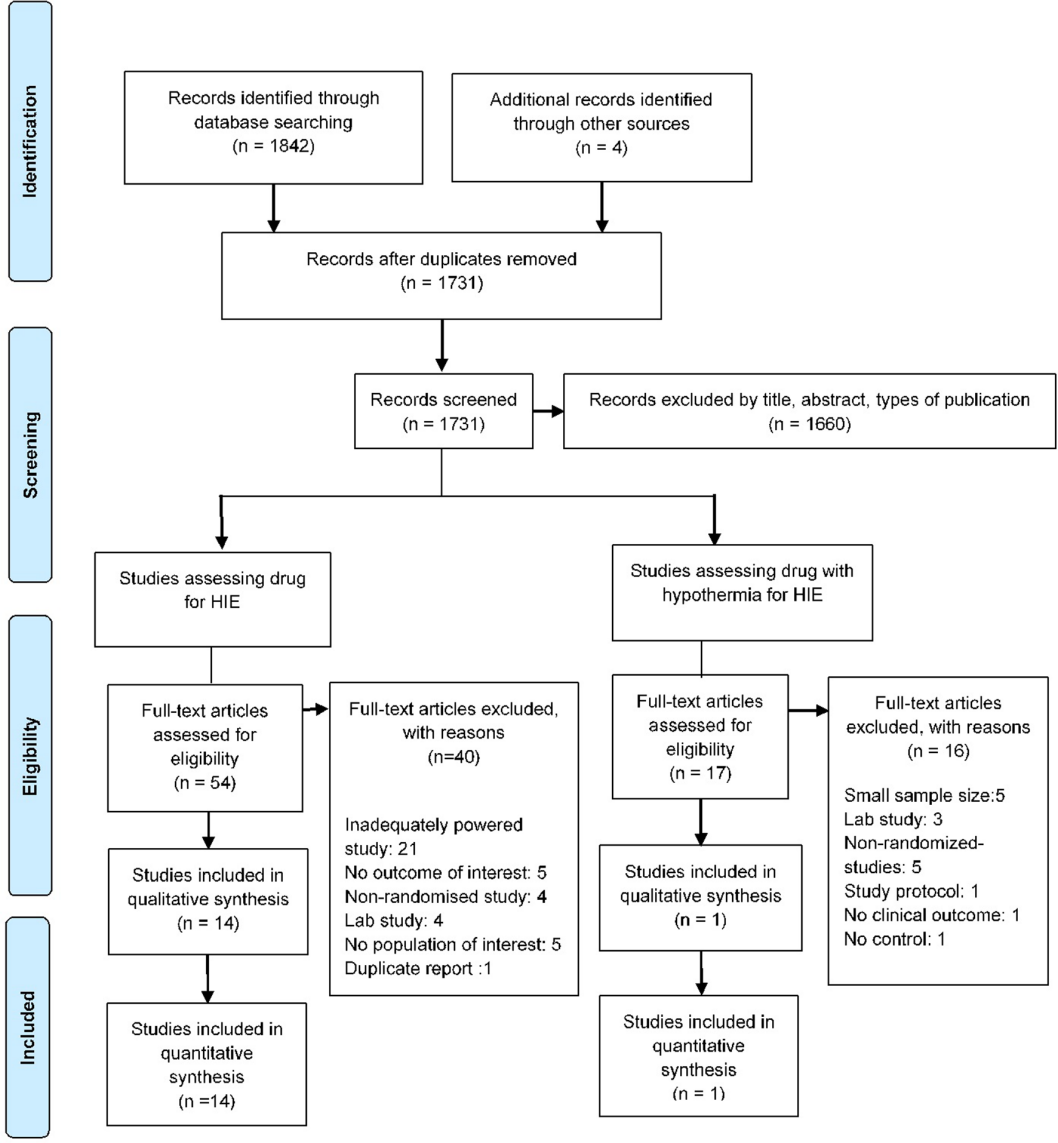
Study flow detailing the screening, identification, and selection process.

The literature search identified a total of 1,731 studies and 71 full-text articles were assessed for eligibility (**Figure 1** and **eTables 1** and **2**). Fifteen studies enrolling 2,313 newborns were included in the review (Gluckman et al., 2005; Shankaran et al., 2005; Li et al., 2009; Zhu et al., 2009; Simbruner et al., 2010; Zhou et al., 2010; Joy et al., 2013; Azzopardi et al., 2016; Savitha and Prakash, 2016; Malla et al., 2017; Sreenivasa et al., 2017). Most of the included studies had a similar enrollment criteria and included infants with evidence of birth asphyxia as defined by the American College of Obstetrician and Gynecologists (American College of Obstetricians and Gynecologists, and American Academy of Pediatrics, 2014) with moderate to severe HIE. These studies had recruited newborns which were at least 35 weeks in 1 study, at least 36 weeks in 3 studies, and gestation weeks of more than or equal to 37 weeks in 9 studies. Interventions examined by studies were therapeutic hypothermia examined in 10 studies (8 whole body cooling and 2 selective head cooling), magnesium sulfate in 2 studies, erythropoietin in 2 studies, and use of xenon with therapeutic hypothermia in 1 study (**Table 1**). Eight studies (53.3%) were published in 2011 or later, and the studies were conducted in India (40%), China (20%), or were multicentered studies (20%). Nine studies (60%) had a duration (from recruitment to end of follow-up) of at least 18 months or more.

**Table 1:** Characteristics of included studies.

		First Author, Year (Study)	Study Population	Interventions	Number of Patients Enrolled	Mean gestational age or age range, weeks
**Drug Intervention as Single Treatment**	**Erythropoietin**	Malla et al., 2017	Neonates (≥37 weeks) with moderate or severe HIE (10 min Apgar score <5) evidence of fetal distress, need for resuscitation at 10 mins after birth	**I:** 500 U/kg rhEPO intravenously on alternate dose for a total of 5 doses with the 1^st^ dose within 6 hours after birth **C:** 2mL of normal saline	100	39.5
		Zhu et al., 2009	Term neonates (≥37 weeks), body weights >2500 g with evidence of perinatal HIE (5-min Apgar score ≤5, need for resuscitation at 10 mins after birth)	**I:** 300 U/kg or 500 U/kg rhEPO subcutaneously for 1^st^ dose and intravenously every other day for 2 weeks **C:** Conventional treatment	167	37.5
	**Magnesium**	Savitha and Prakash, 2016	Term neonates with perinatal asphyxia, 1-min Apgar score < 7, need for resuscitation at birth, failure to initiate breath at birth	**I:** 250 mg/kg MgSO_4_ intravenous infusion over 1 h within 6 h of birth, with additional doses repeated at 24 h and 48 h **C:** Supportive care	120	38.5
		Sreenivasa et al., 2017	Term neonates with perinatal asphyxia, 1-min Apgar score <3 or 5-min Apgar score <6	**I:** 250 mg/kg MgSO_4_ intravenous infusion over 1 h within 6 h of birth, with additional doses repeated at 24 h and 48 h **C:** Supportive care	100	38.7
	**Cooling (Whole body)**	Azzopardi et al., 2009 (TOBY)	Term neonates (≥36 gestation weeks) with moderate to severe HIE, Apgar score <6, seizure on aEEG	**I:** Manually adjusted cooling blanket to target rectal temperature of 33.0°C–34.0°C for 72hr **C:** Conventional care with overhead radiant heater to target rectal temperature of 36.8°C–37.2°C	325	38.8–41.3^†^
		Bharadwaj, 2012	Term neonates (>37 gestation weeks) with perinatal asphyxia (10-min Apgar score ≤6), and encephalopathy	**I:** Whole body cooling with gel packs to target rectal temperature of 33.0 °C–34.0°C **C:** Conventional care with servo-controlled overhead radiant heater to target rectal temperature of 36.5°C	130	40.0
**Drug Intervention as Single Treatment**	**Cooling (Whole body)**	Gane, 2013	Term neonates (≥37 weeks) with evidence of encephalopathy (10-min Apgar <5)	**I:** Cloth covered gel packs to target rectal temperature of 33°C–34°C for 72 h **C:** Conventional care for HIE to target of 36.5°C	122	40.1
		Jacobs et al., 2011 (ICE)	Term or near term neonates (≥35 gestation weeks) with moderate to severe HIE, perinatal asphyxia, 10-min Apgar score <6	**I:** Refrigerated gel pack across chest and/or under head and shoulders to target rectal temperature of 33.0°C–34.0°C for 6–72 h **C:** Conventional care with overhead radiant heater to target rectal temperature of 36.8°C –37.2°C	221	39.1
		Joy et al., 2013	Term neonates (≥37 weeks) with evidence of encephalopathy (10-min Apgar ≤5)	**I:** Cloth covered gel packs to target rectal temperature of 33–34 °C for 72 h **C:** Conventional care for HIE to target rectal temperature of 36.5°C	160	-
		Li et al., 2009	Term neonates (≥37 weeks), weight > 2500 g, with moderate to severe encephalopathy (5-min Apgar ≤5)	**I:** Whole body cooling with cooling mattress to target rectal temperature of 33 °C–34 °C for 72 h **C:** Conventional care for HIE to target rectal temperature of 36.5°C–37.5 °C	93	39.1
		Shankaran et al., 2005 (NICHD study)	Term neonates (≥37 gestation weeks) with moderate to severe HIE, perinatal asphyxia (10 min Apgar score ≤5))	**I:** Two servo-controlled cooling blanket to target oesophageal temperature of 34.5 °C for 72 h **C:** Conventional care with overhead radiant heater to target skin temperature of 36.5 °C–37.0 °C	208	4.3 h*
		Simbruner et al., 2010 (neo,nEURO)	Term neonates (≥36 gestation weeks) with moderate to severe HIE, perinatal asphyxia (10 min Apgar score <5) and encephalopathy as evidence by abnormal standard EEG or aEEG findings	**I:** Cooling blanket to target rectal temperature of 33.0°C–34.0°C for 72 h **C:** Conventional care to target rectal temperature of 36.5°C–37.5°C	129	39.3
	**Cooling (Selective head)**	Gluckman et al., 2005 (CoolCap)	Term neonates (≥37 gestation weeks) with moderate to severe HIE, perinatal asphyxia, 10-min Apgar score ≤5, severe acidosis (pH < 7) or a base deficit of 16 mmol/L	**I:** Manual controlled cooling cap to target temperature of 34.0°C –35.0°C for 72 h **C:** Conventional care with overhead radiant heater to target of 36.5°C –37.5°C	234	39.0
		Zhou et al., 2010	Term neonates (≥37 gestation weeks) with perinatal asphyxia (5-min Apgar score ≤5 or 1-min Apgar score ≤3), birth weight ≥2500 g, and encephalopathy	**I:** Manually controlled cooling cap to rectal target temperature of 34.5°C –35.0°C for 72 h **C:** Conventional care whereby infants are cared on radiant warmers servo-controlled to rectal target of 36.0°C–37.5°C	194	4.0 h*
**Drug Intervention as Adjuvant**	**Xenon**	Azzopardi et al., 2016 (TOBY-Xe)	Gestation weeks (36–43 weeks), had signs of moderate to severe encephalopathy, moderately or severely abnormal background activity for ≥30 min or seizures shown by aEEG, 10-min Apgar score ≤5, continued need for resuscitation for ≥10 min	**I:** Whole body hypothermia to target rectal temperature of 33.5°C plus 30% inhaled xenon for 24 h **C:** Whole body hypothermia alone to target rectal temperature of 33.5°C	92	39.8

AAP, American Association of Pediatrics; ACOG, American College of Obstetricians and Gynecologists; aEEG, amplitude-integrated continuous electroencephalopathy; C, control group; CBV, cerebral blood volume; EAA, excitatory amino acids; EPO, erythropoietin; 1^st^, first; I, intervention group; h, hours; HIE, hypoxic-ischemic encephalopathy; MgSO_4_, magnesium sulfate; min, minutes; m, months; NaCl, normal saline; NO, nitric oxide; rhEPO, recombinant human erythropoietin; subcut, subcutaneously.

* Age at randomization; ^†^Interquartile range.

### Methodological Quality of Included Studies

Twelve (80.0%) studies had adequate reporting on sequence generation, 12 (80.0%) studies described the loss to follow-up, 10 (66.7%) studies described the selection concealment adequately, and 6 (40.0%) studies described the blinding of participants and outcome assessment. However, a high proportion [10 (66.7%)] had unclear risk of bias for blinding of outcome assessors (**eFigure 1**).

### Primary Outcomes

#### Mortality

Fifteen RCTs reported the effectiveness of intervention in reducing the risk of mortality. Pairwise meta-analysis showed that whole body hypothermia was effective in reducing the risk of mortality (OR: 0.71; 0.52–0.92, *I* ^2^ = 0%) compared to usual care (**eTable 3**).

#### Mortality and/or Neurodevelopmental Delay at 18 Months 

Nine studies reported the long term effects of pharmacotherapy or hypothermia on mortality and neurodevelopmental delay at 18 months (Gluckman et al., 2005; Shankaran et al., 2005; Azzopardi et al., 2009; Li et al., 2009; Zhu et al., 2009; Simbruner et al., 2010; Zhou et al., 2010; Jacobs et al., 2011; Malla et al., 2017). Pooled analysis of studies suggest that both erythropoietin (OR: 0.57; 0.36–0.91, *I* ^2^ = 0%) and whole body hypothermia (0.74; 0.59–0.92, *I* ^2^ = 0%) were effective in reducing the risk of mortality and neurodevelopmental delay at 18 months.

#### Secondary Outcomes

Among infants who survived, 12 trials including 1,951 infants reported on cerebral palsy outcome. Whole body hypothermia was found to be statistically superior in reducing the odds developing cerebral palsy (0.70; 0.54–0.92, *I* ^2^ = 0%) compared to usual care. With respect to seizures, 10 trials including 1,703 infants were included. Whole body hypothermia was superior in reducing the rates of seizure compared to usual care (0.73; 0.56–0.96, *I* ^2^ = 0%). 

Five studies reported the neuromotor delay and neurodevelopmental delay among neonates using the Bayley II index, but results were not significant. For the other outcomes of renal failure, sepsis, hypotension, hypoglycemia, bradycardia, hearing loss, and blindness, the evidence base were sparse (**eTable 4**). Meta-analysis suggests that therapeutic hypothermia (both whole body and selective head cooling) were associated with an increased rate of normal survival, defined as survival without cerebral palsy, seizures, normal vision, and hearing. 

### Effect of Severity of Encephalopathy

Most studies assessed the severity of encephalopathy by clinical assessment using Sarnat’s criteria (Sarnat and Sarnat, 1976), which classifies the degree of encephalopathy to either stages I, II or III, correlating with mild, moderate or severe encephalopathy. Pooled analysis suggests that the relative odds of mortality or neurodevelopmental disability were lower among infants with moderate encephalopathy when treated with erythropoietin (0.27; 0.11–0.63, *I* ^2^ = 0%) or whole body hypothermia (0.63; 0.44–0.89, *I* ^2^ = 0%). Among infants with severe encephalopathy, all interventions including erythropoietin, whole body hypothermia or selective head hypothermia was ineffective in reducing the risk of combined outcomes of mortality or neurodevelopmental disability (**eTable 5**). 

### Network Meta-Analysis

#### Mortality or Mortality and Neurodevelopmental Disability at 18 Months

Overall, of the 15 unique pairwise comparisons that could be made, only 5 were studied head to head. The network meta-analysis gave an adequate fit to the data and design-by-treatment model showed no evidence of inconsistency (**eFigure 2**).

**Figure 2:**
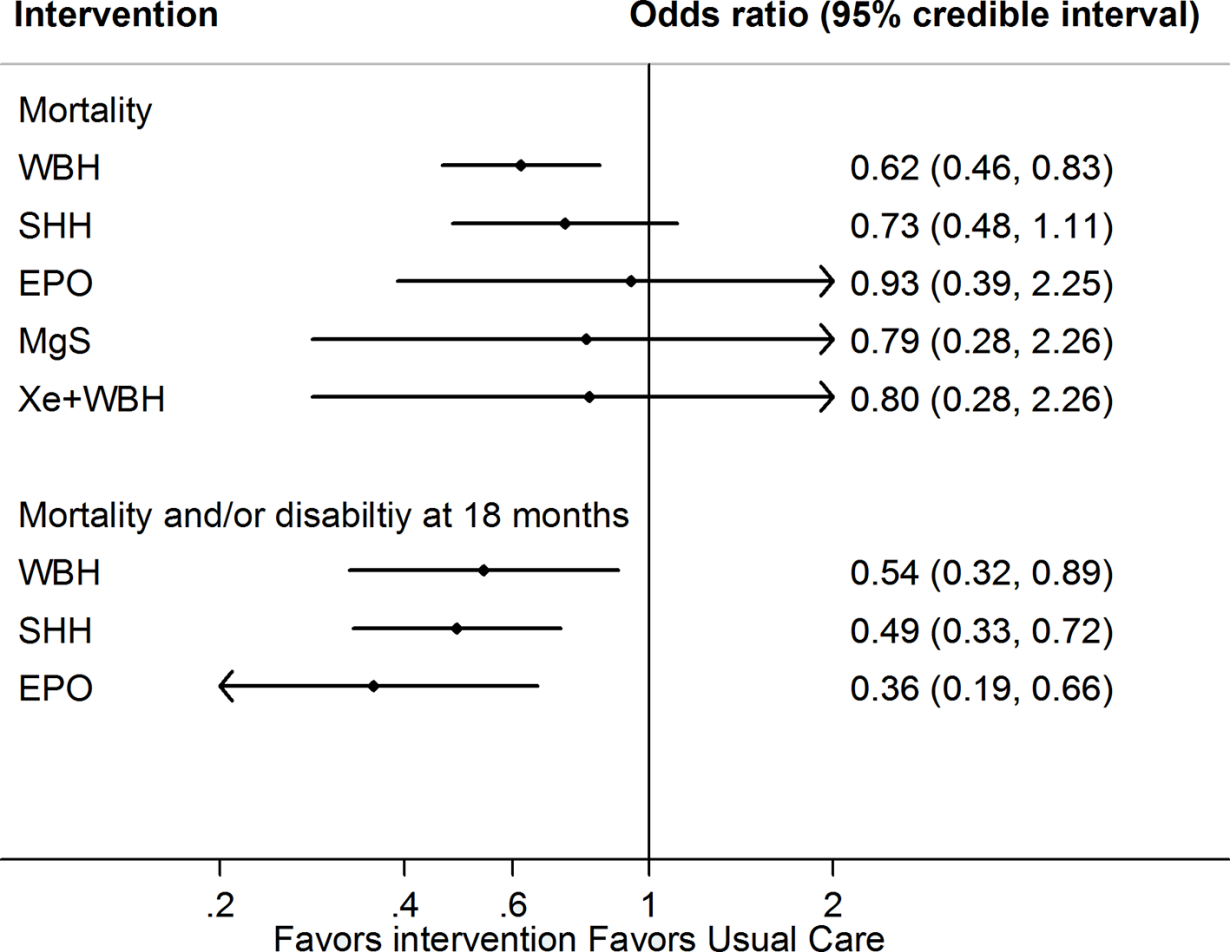
Network meta-analysis forest plots for each treatment versus usual care on mortality or mortality and neurodevelopmental delay at 18 months outcome. Each rhombus represents the summary treatment effect estimated in the network meta-analysis on the odds ratio (OR) scale. The black horizontal lines represent the credible intervals (CrI) for the summary treatment effects; an OR > 1 suggests that usual care is more effective to reduce the risk of mortality, whereas an OR < 1 suggests that the comparable treatment is better. The vertical blue line corresponds to an OR = 1.

All interventions examined showed a reduction in risk of mortality compared to usual care (**Figure 2**, **Table 2** and **eTable 6**). However, only whole body hypothermia was significantly better than usual care (0.62; 0.46–0.83), with an NNT of 11 (95% CI: 7–26) in reducing the risk of mortality (**eTable 7**). With respect to the composite mortality or neurodevelopmental disability outcome at 18 months or longer, erythropoietin was significantly better than usual care in patients with moderate encephalopathy (0.36; 0.19–0.66; **eFigure 3**). Therapeutic hypothermia (both whole body and selective head cooling) was the only treatment statistically superior to usual care in patients with moderate (whole body: 0.45; 0.31–0.66 and selective head: 0.51; 0.29–0.89) or severe encephalopathy (whole body: 0.32; 0.12–0.86).

**Table 2:** Network meta-analysis for primary outcomes mortality.

**Whole body hypothermia**	1.11 (0.59, 2.08)	0.66 (0.26, 1.67)	0.78 (0.20, 3.14)	0.77 (0.29, 2.09)	0.62 (0.46, 0.83)
	**Selective head hypothermia**	0.78 (0.30, 2.07)	0.92 (0.22, 3.83)	0.91 (0.30, 2.79)	0.73 (0.48, 1.11)
		**Erythropoietin**	1.18 (0.24, 5.96)	1.17 (0.30, 4.55)	0.93 (0.39, 2.25)
			**Magnesium sulfate**	0.99 (0.18, 5.45)	0.79 (0.20, 3.06)
				**Whole body ** **hypothermia with ** **xenon**	0.80 (0.28, 2.26)
					**Usual care**

Comparisons should be read from left to right. The estimate is located at the intersection of the column-defining treatment and the row-defining treatment. An OR value below 1 favors the column-defining treatment. To obtain ORs for comparisons in the opposing direction, reciprocals should be taken. Any significant results are in bold and underlined.

#### Secondary Outcomes

**Figure 3:**
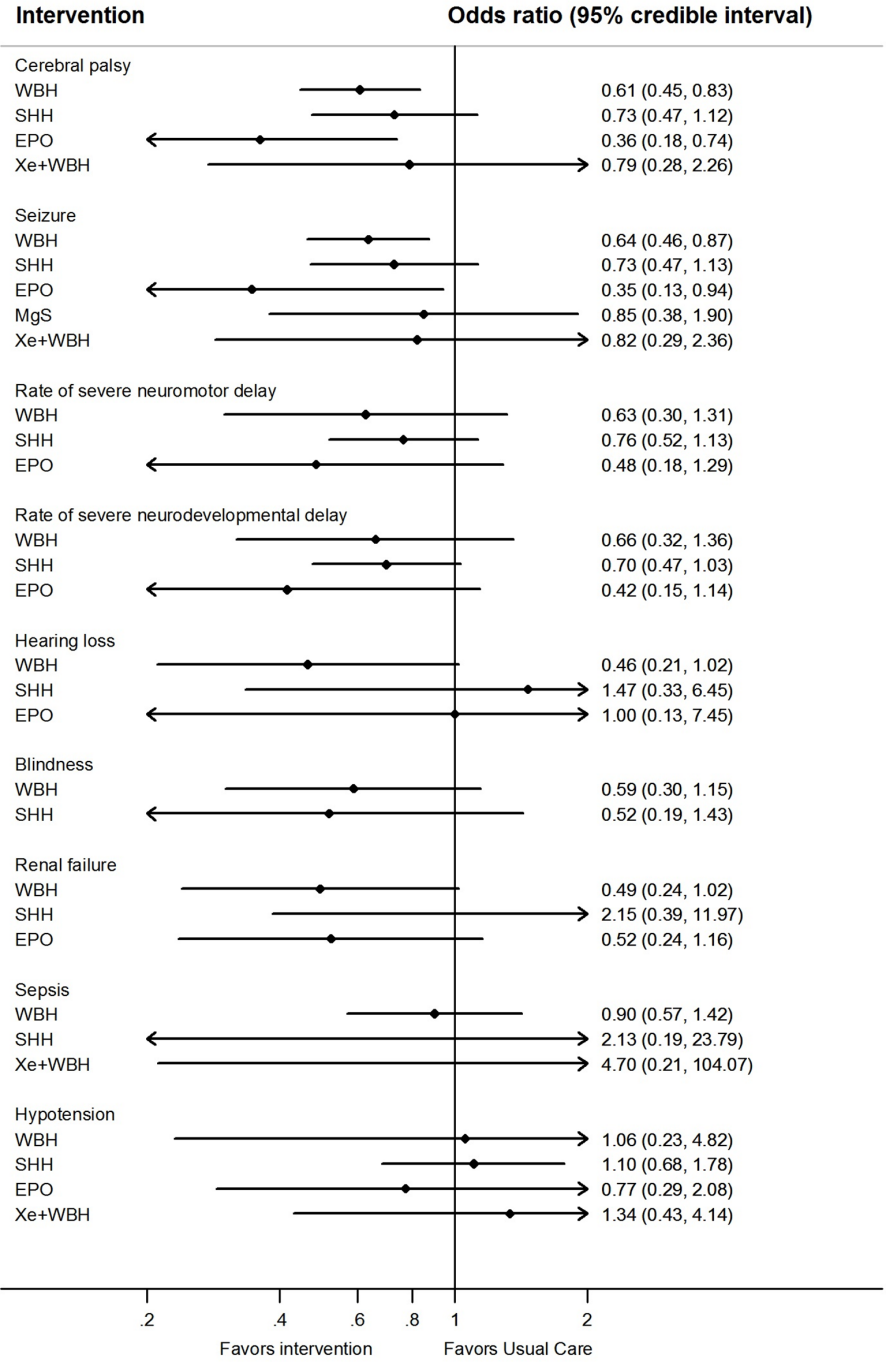
Network meta-analysis forest plots for each treatment versus usual care on secondary outcomes. Each rhombus represents the summary treatment effect estimated in the network meta-analysis on the odds ratio (OR) scale. The black horizontal lines represent the credible intervals (CrI) for the summary treatment effects; an odds ratio > 1 suggests that usual care is more effective to reduce the risk of mortality, whereas an OR < 1 suggests that the comparable treatment is better. The vertical blue line corresponds to an OR = 1.

Network meta-analysis suggest that, compared to usual care, treatment with either erythropoietin or whole body hypothermia were associated with lower rates of cerebral palsy (0.36; 0.18–0.74 and 0.61; 0.45–0.83, respectively) and seizures (0.35; 0.13– 0.94 and 0.64; 0.46–0.87, **Figure 3**).

#### Sensitivity Analyses

Comparison-adjusted funnel plots of the network meta-analysis for primary outcomes did not suggest any publication bias (**eFigure 4**). Ranking of treatment based upon cumulative probability plots (**eFigure 5**) and SUCRA showed that the most effective treatment for primary outcome mortality was whole body hypothermia (77.8%) and the least effective was usual care (24.5%). In terms of the composite outcome of mortality or neurodevelopmental disability at 18 months, the most effective treatment was erythropoietin (88.8%) and the least effective was usual care (0.2%). Using GRADE, the quality of evidence for primary outcomes were moderate to very low for most comparison (**Table 3** and **eTable 8**). 

**Table 3:** Summary of findings table for the primary outcomes assessed in this study.

Estimates of effects, credible intervals, and certainty of the evidence for comparison of neuroprotective therapies for neonates with hypoxic ischemic encaphalopathy							
Patient or population: Neonates with hypoxic ischemic encephalopathy Interventions: Whole body cooling, selective head cooling, magnesium sulfate, erythropoietin, whole body cooling with xenon Comparator (reference): Usual care Outcome: Mortality; mortality or neurodevelopmental disability at 18 months or later Setting(s): Inpatient and outpatient							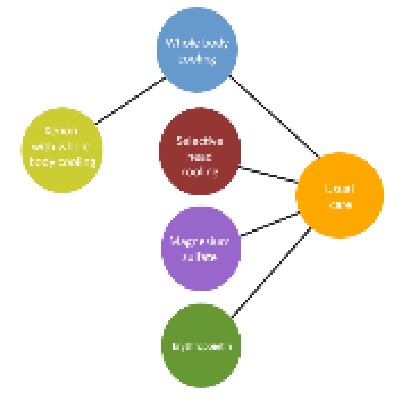 Geometry of the Network
Total studies:15 RCT Total Participants: 2,103		Odds ratio (95% CrI)	Anticipated absolute effect (95% CrI)			Certainty of the evidence	Interpretation of Findings
			With placebo	With intervention	Difference		
Mortality							
•	Whole body cooling (Direct evidence; 8 RCT; 1324 participants)	**0.62** (0.46 to 0.83)	261 per 1,000	180 per 1,000	81 fewer per 1,000 (from 121 fewer to 34 fewer)	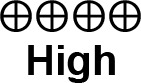	Whole body cooling improves survival in newborns with HIE
•	Selective head cooling (Direct evidence; 2 RCT; 428 participants)	**0.73** (0.48 to 1.11)	259 per 1,000	204 per 1,000	56 fewer per 1,000 (from 115 fewer to 21 more)	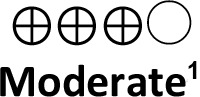	Selective head cooling probably improves survival in newborns with HIE
•	Magnesium sulfate (Direct evidence; 2 RCT; 220 participants)	**0.79** (0.20 to 3.06)	36 per 1,000	29 per 1,000	7 fewer per 1,000 (from 29 fewer to 67 more)	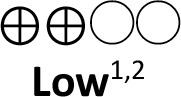	Use of magnesium sulfate has limited effect on survival in newborns with HIE
•	Erythropoietin (Direct evidence; 2 RCT; 253 participants)	**0.93** (0.39 to 2.25)	89 per 1,000	84 per 1,000	6 fewer per 1,000 (from 53 fewer to 92 more)	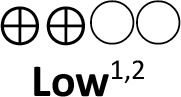	Use of erythropoietin has limited effect on survival in newborns with HIE
•	Whole body cooling with xenon (Indirect evidence; 1 RCT; 92 participants)	**0.80** (0.28 to 2.26)	196 per 1,000	163 per 1,000	33 fewer per 1,000 (from 132 fewer to 159 more)	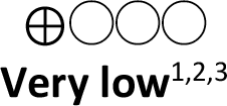	Use of xenon as an adjuvant with whole body cooling has limited effect on survival in newborns with HIE
Mortality or neurodevelopmental delay at 18 months							
•	Whole body cooling (Direct evidence; 5 RCT; 934 participants)	0.54 (0.32 to 0.89)	607 per 1,000	455 per 1,000	152 fewer per 1,000 (from 276 fewer to 28 fewer)	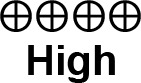	Whole body cooling improves survival and neurodevelopment in newborns with HIE
•	Selective head cooling (Direct evidence; 2 RCT; 412 participants)	**0.49** (0.33 to 0.72)	583 per 1,000	407 per 1,000	176 fewer per 1,000 (from 267 fewer to 81 fewer)	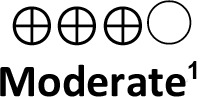	Selective head cooling probably improves survival and neurodevelopment in newborns with HIE
•	Erythropoietin (Direct evidence; 2 RCT; 253 participants)	**0.36** (0.29 to 0.67)	538 per 1,000	296 per 1,000	243 fewer per 1,000 (from 286 fewer to 100 fewer)	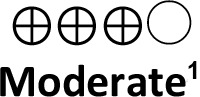	Use of erythropoietin probably improves survival and neurodevelopment in newborns with HIE

^1^Study was downgraded due to imprecision and lack of direct RCTs contributing to direct evidence; ^2^Few event rates with wide confidence intervals leading to imprecision; ^3^Serious indirectness; ^4^Contributing direct evidence of moderate quality with inadequate concealment of allocation and blinding.

In the preplanned sensitivity analyses, we excluded studies which were conducted in low-middle income countries since previous meta-analysis have suggested that these study setting could influence the results (Pauliah et al., 2013). When studies from high-income countries were only included, only three comparisons were possible for the outcome of mortality. Results from the network meta-analysis was largely unchanged, and whole body cooling was found to reduce the risk of mortality by 36% (0.64; 0.46–0.90) as well as mortality or major neurodevelopmental disability at 18 months by 49% (0.51; 0.33–0.78) compared to usual care. These results were largely unchanged when we included studies which had examined full term neonates (≥37 weeks), where whole body cooling reduced the risk of mortality (0.50; 0.31–0.81) as well as mortality or major neurodevelopmental disability at 18 months (0.44; 0.27–0.71) compared to usual care (**eTable 9**). In addition, selective head cooling was also noted to lower the risk of mortality or major neurodevelopmental disability at 18 months compared to usual care (0.54; 0.36–0.81).

## Discussion

In this study, direct and indirect evidence from 15 RCTs was combined to compare the association of each therapy used for neuroprotection in HIE. The study has several key findings. Firstly, therapeutic hypothermia was significantly associated with lower odds of mortality and neurodevelopmental delays compared to usual care, with high confidence estimates and a number to treat of between 11 and 16. Secondly, whole body hypothermia should be offered to all infants with HIE, irrespective of the severity as it was effective in reducing the risk of morbidity as well as neurodevelopmental delays when compared to usual care. 

Over the past decade, numerous drugs have been proven useful to be beneficial in animal models for neonates with HIE, but how these can be translated into clinical use remains unknown (Nair and Kumar, 2018). Our study provides novel insights into how different neuroprotective agents could be useful in patients with HIE especially when used in combination with hypothermia (Martinello et al., 2017). Although there is much progress, further studies are needed to determine the effectiveness of these adjuvant therapies. Several large clinical studies are underway to examine the benefits of these neuroprotective agents. 

A previous review suggested that key differences exists in terms of efficacy between high-income and low-middle income countries, due to the use of low-technology devices, degree of encephalopathy, maternal pre-existing diseases, malnutrition status, infections, as well as study inclusion criteria (Pauliah et al., 2013). In this study, we showed that therapeutic hypothermia especially whole body hypothermia was the most effective intervention irrespective of study setting as well as device used. As HIE is the major cause of up to 23% of 2.8 million neonatal deaths especially in low-resource setting, our findings provide a further impetus for therapeutic hypothermia to be part of standard of care especially in low-middle income countries. 

Our findings support the Cochrane review on hypothermia in patients with HIE for the primary outcome, where we found that cooling was beneficial in reducing the risk of mortality and disability (Jacobs et al., 2013). This has similarly been reported by other authors which concluded that hypothermia improves survival and neurodevelopmental delays in newborns (Tagin et al., 2012; Douglas-Escobar and Weiss, 2015). The current review was larger (an additional 4 RCTs and 808 infants) and includes information on the effectiveness of different neuroprotective agents. While the authors of a recent systematic review on hypothermia did not conduct network meta-analysis, the results presented were similar to those reported here and suggest that therapeutic hypothermia is effective. 

### Study Strength

Strengths of our review are that we conducted a comprehensive search as well as the identification of new additional studies. We also used multiple approaches to assess the relationship of effects and performed a network meta-analysis, which provides added information on effects of different combination of interventions (drugs and non-drugs). The quality of the evidence generated were rated using the GRADE criterion (Puhan et al., 2014). We used a comprehensive search strategy and searched all pertinent sources for eligible studies, which reduces the possibility of missing any relevant studies. This study also included preterm infants ≥35 weeks as part of the inclusion criteria and provides a more holistic overview on the clinical safety and efficacy of therapeutic hypothermia in this group of infants where data on outcomes are sparse. Nevertheless, these results need to be confirmed from larger randomized controlled trials such as the Premmie Hypothermia for Neonatal Encephalopathy study which is currently in progress (NCT01793129). Until then, clinicians should take precaution especially when treating preterm neonates since evidence from a recent retrospective cohort analysis have suggested that a high incidence of complication and composite outcome of death and neurodevelopmental impairment (Herrera et al., 2018).

### Study Limitations

The limitations of our review are the inherent heterogeneity in terms of study design, intervention, as well as outcome assessment of included studies. We attempted to minimize this by using rigorous selection criteria and performing several sensitivity analyses to ensure the robustness of our results. Secondly, analysis for other outcomes such as neurodevelopmental outcomes, blindness, as well as adverse events should be interpreted with caution, owing to the few data points available. However, a recent high quality long-term study on effects of depth and duration of cooling showed that there is little to no effect of different hypothermia therapy or duration of therapy on outcome (Shankaran et al., 2014; Laptook et al., 2017). This independent analysis reinforces the case that hypothermia should be standard therapy, and additional policy options may be needed in low resource settings to improve outcomes. As with most network meta-analyses, there were only sparse data for some of the treatment comparison especially those related to erythropoietin, and thus, it is recommended that these treatment effect estimates be interpreted together with their precision. 

Our study also revealed that additional studies are needed to further optimize cooling therapy as well as rewarming methods, such as the recently concluded NICHD funded study which examined the impact of different cooling depth and duration in neonates with HIE (Shankaran et al., 2014; Laptook et al., 2017). In addition, sufficiently powered studies which examine the use of adjuvant therapies in addition to hypothermia are needed. Another research goal is to reliably identify for subgroups of newborns who will go on to develop worsening encephalopathy and significant brain injury, possibly through the use of electroencephalography, or other biomarker to ensure that they benefit most from hypothermia. In summary, results of our analysis generally support current guidelines using hypothermia for neonates with HIE irrespective of setting. Our findings further support whole body hypothermia as first line, due to its ease of use, improving mortality and neurodevelopmental outcomes. However, further research is needed to determine if the use of additional adjuvant therapies could further improve outcomes of HIE. 

## Data Availability Statement

All datasets generated and analysed in this study are included in the article/**Supplementary Material**.

## Author Contributions

SL conceptualized the study, designed the data collection instrument, carried out the statistical analysis, drafted the initial manuscript, and reviewed and revised the manuscript. CL and PC carried out the data acquisition and helped drafted out the initial manuscript. All authors approved the final manuscript as submitted and agree to be accountable for all aspects of the work.

## Conflict of Interest

The authors declare that the research was conducted in the absence of any commercial or financial relationships that could be construed as a potential conflict of interest.

The handling editor and reviewer, BG, declared their involvement as co-editors in the Research Topic, and confirm the absence of any other collaboration.

## Acnowledgments

We wish to thank Dr. Nai Ming Lai of Taylor’s University for helping edit the manuscript for clarity, Dr. Mohamed Tagin of University of Toronto, and Dr. Seetha Shankaran of Wayne State University School of Medicine for providing input and advice on this article. 
