# Supplementary material for: Comparative Efficacy and Safety of Neuroprotective Therapies for Neonates With Hypoxic Ischemic Encephalopathy: A Network Meta-Analysis
Source: Front Pharmacol. 2019 Oct 25;10:1221. doi: 10.3389/fphar.2019.01221 (PMC6824259; doi:10.3389/fphar.2019.01221)

# Supplement

**Search algorithms**

We searched PUBMED using the algorithm listed below. The algorithm was adjusted to also search EMBASE, CINAHL Plus, Allied and Complementary Medicines (AMED) and Cochrane Library.

| **Database** | **Search algorithms** | |
| --- | --- | --- |
| **PUBMED** | #1 | asphyxia |
|  | #2 | asphyxia neonatorum |
|  | #3 | hypoxic ischemic encephalopathy |
|  | #4 | ischemic encephalopathy |
|  | #5 | brain ischemia |
|  | #6 | #1 OR #2 OR #3 OR #4 OR #5 |
|  | #7 | magnesium |
|  | #8 | magnesium sulfate |
|  | #9 | deferoxamine |
|  | #10 | deferoxamine mesylate |
|  | #11 | cannabinoids |
|  | #12 | cannabidiol |
|  | #13 | marijuana |
|  | #14 | melatonin |
|  | #15 | statin |
|  | #16 | N-acetyl-5-methoxytryptamine |
|  | #17 | topiramate |
|  | #18 | xenon |
|  | #19 | allopurinol |
|  | #20 | erythropoietin |
|  | #21 | iminobiotin |
|  | #22 | N-acetylcysteine |
|  | #23 | barbiturate |
|  | #24 | #7 OR #8 OR #9 OR #10 OR #11 OR #12 OR #13 OR #14 OR #15 OR #16 OR #17 OR #18 OR #19 OR #20 OR #21 OR #22 OR #23 |
|  | #25 | infant |
|  | #26 | newborn |
|  | #27 | neonate |
|  | #28 | #25 OR #26 OR #27 |
|  | #30 | #6 AND #24 |
|  | #31 | #30 AND 28 |

**eTable 1: I**nclusion criteria of included studies

| **First Author,**  **Year** | **Definition of asphyxia** | **Severity of HIE** | **Presence of seizure** |
| --- | --- | --- | --- |
| Azzopardi, 2009 | 10-min Apgar <5 and/or continued need for resuscitation, blood acidosis (umbilical-cord, arterial or capillary pH <7 or base deficit >16mmol/L | Signs of lethargy, stupor or coma | Amplitude-integrated electro-encephalography |
| Azzopardi, 2015 | 10-min Apgar <5, continued need for resuscitation including endotracheal or mask ventilation, blood acidosis (umbilical cord or any blood sample pH<7 or base deficit >15mmol/L, or both) | Signs of altered state of consciousness (reduced or absent response to stimulation), hypotonia or severe hypotonia, abnormal primitive reflexes (weak or absent suck or Moro response) | Amplitude-integrated electro-encephalography |
| Bharadwaj, 2012 | Arterial blood gas pH*<*7 or base excess >12 meq within the 1^st^ hr after birth and any two of the following criteria: (1) 10-min Apgar <6 , (2) evidence of foetal distress; (3) assisted ventilation  for at least 10 min after birth; (4) evidence of any organ dysfunction; and (5) history of acute perinatal event like intrapartum foetal distress, cord prolapsed, placental abruption, uterine rupture and maternal trauma | Sarnat stage 2-3 | - |
| Gane 2014 | 10-min Apgar <5, with umbilical cord blood or arterial cord blood (within 1^st^ postnatal hr) pH <7 or base deficit >16meQ | Evidence of fetal distress, assisted ventilation for at least 10 mins after birth, evidence of organ dysfunction | - |
| Gluckman, 2005 | 10-min Apgar <5, continued need for  resuscitation, including endotracheal or mask ventilation at 10 min after birth; or severe acidosis (pH <7·00 or a base deficit >16 mmol/L in an umbilical cord blood sample or an arterial or venous blood sample obtained within 60 min of birth | Pre-randomsation EEG or Sarnat stage 2-3 | Amplitude-integrated electro-encephalography |
| Jacobs, 2011 | 2 of the following clinical characteristics: 10-min  Apgar <5, continued need for mechanical  ventilation at 10 minutes, and/or metabolic acidosis  (cord pH_7.00; an arterial, venous, or capillary pH_7.00; or a base deficit >12 within 60 minutes of birth). | Modified Sarnat critaria | - |
| Joy, 2013 | Umbilical cord blood or peripheral blood pH<7  or base deficit >12mEq within 1hr of birth, and  any two of the following criteria) 10-min Apgar <5 , (2) assisted ventilation initiated at birth for at least 10 min after birth, (3) evidence of any organ dysfunction, (4) history of acute perinatal event (intrapartum fetal distress, cord 6rolapsed, placental  abruption, uterine rupture, maternal trauma  or cardiac arrest) | Modified Sarnat critaria | - |
| Li, 2009 | 5-min Apgar <5 or continued need for resuscitation (use of endotracheal or mask ventilation); pH<7.1 or base deficit >16mmol/L within 1 hr | Sarnat’s criteria | - |
| Malla, 2017 | 10-min Apgar <5 and two of the following criteria: (1) fetal distress (late deceleration or loss of beat-to-beat variability or fetal bradycardia or meconium-stained amniotic fluid)., (2) need for immediate neonatal ventilation with a bag and mask or through endotracheal intubation for ⩾10 min after delivery, (3) Base deficit of ⩾ 16 mEq/L and/or pH U7.0 in cord blood or arterial blood samples within 1hr after birth | Presence of 3 of the categories:  (1) Level of consciousness: lethargy or stupor or coma, (2) Spontaneous activity: decreased or absent, (3) Tone: hypotonia or flaccidity, (4) Posture: distal flexion or decerebrate state, (5) Primitive reflexes: suck weak or absent or Moro reflex incomplete or absent, (6) Autonomic nervous system: pupils constricted or deviated, dilated or non-reactive to light, bradycardia or variable heart rate, or periodic breathing or apnea | - |
| Savitha 2016 | Failure to initiate breath or 1-min Apgar <7 and need for resuscitation at birth (positive pressure ventilation or chest compression) | Sarnat’s criteria | - |
| Shankaran 2005 | pH<7 or base deficit >16 mmol or (if no blood gas or pH7.01-7.15) or acute perinatal event and seizure | Modified Sarnat stage 2-3 | - |
| Simbruner, 2010 | 10 min Apgar <5 ; cord blood pH <7 or base deficit >16 mmol; continued need for resuscitation at 10 mins after birth | - | Amplitude-integrated electro-encephalography |
| Sreenivasa, 2017 | 1-min Apgar <3 or 5-min Apgar<6; | Sarnat’s criteria | - |
| Zhou, 2010 | 1-min Apgar <3 or 5-min Apgar <5, cord blood gas pH <7.0 or base deficit <16 mmol/L; and need for resuscitation or ventilation at 5-min of age | Sarnat’s criteria | - |
| Zhu 2009 | 5-min Apgar <5, continued need for resuscitation (including endotracheal or mask ventilation) | Sarnat’s criteria | - |

**eTable 2: Excluded studies with reasons**

|  |  | **First Author, Year** | **Reason for exclusion** |
| --- | --- | --- | --- |
| **Drug Intervention as Single Treatment** | **Allopurinol** | Benders, 2006 | Small study with sample size of only 32 participant |
|  |  | Gunes, 2007 | Mechanistic study |
|  |  | Kaandorp JJ, 2010 | Prenatal allopurinol received by pregnant women. |
|  |  | Kaandorp JJ, 2013 | Allopurinol given to pregnant mothers. |
|  |  | Torrance HL, 2009 | Allopurinol given to pregnant mothers. |
|  |  | Van Bel, 1998 | Mechanistic study |
|  | **Melatonin** | Fulia F, 2001 | Clinical biochemistry reported. No outcome of interest. |
|  | **Erythropoietin** | Avasiloaiei, 2013 | Insufficiently powered study |
|  |  | Fauchere JC, 2015 | Infants with hypoxic ischemic encephalopathy were not in inclusion criteria of the study. |
|  |  | Elmahdy, 2010 | Small study with sample size of only 30 participant |
|  |  | El Shimi MS, 2014 | Case-control study. |
|  |  | Wang 2011 | Unclear diagnosis of moderate to severe HIE |
|  | **Magnesium** | Bhat, 2009 | Study had a small sample |
|  |  | Dunne JT, 1971 | Animal study. |
|  |  | Evans, 1996 | Suggested study design but no data published. |
|  |  | Gathwala G, 2006 | Clinical outcomes were not reported. |
|  |  | Gathwala, 2010 | Unclear on study control and small study |
|  |  | Groenendaal, 2002 | Only describes asphyxiated infants but unclear on inclusion |
|  |  | Harrison V, 2007 | Prenatal magnesium received by pregnant women. |
|  |  | Hossain, 2013 | Describes only short term outcome |
|  |  | Ichiba, 2002 | No placebo control |
|  |  | Ichiba H, 2006 | Prospective cohort study. |
|  |  | Khashaba, 2006 | Small sample size which is insufficiently powered |
|  |  | Khashaba MT, 2011 | Lab experiment. No intervention given to human. |
|  |  | Krishna, 2016 | Small sample size which is insufficiently powered |
|  |  | Levene M, 1995 | Methodology was not clarified despite contacted the author due to data protection (data destroyed). |
|  |  | Maroszynska I, 1999 | All infants were in intervention group. |
|  |  | Mahmood, 2015 | Small sample size which is insufficiently powered |
|  |  | Szemraj J, 2006 | Clinical biochemistry reported. |
|  | **Whole body hypothermia** | Atici, 2015 | Small sample size which is insufficiently powered |
|  |  | Eicher, 2005 | Small sample size which is insufficiently powered |
|  |  | Maoulainine, 2017 | Non-randomized study with incompatible control group |
|  |  | Robertson, 2008 | Small sample size which is insufficiently powered |
|  |  | Shankaran, 2002 | Mechanistic study |
|  |  | Thayyil, 2013 | Small sample size which is insufficiently powered |
|  | **Selective head hypothermia** | Akisu, 2003 | Small sample size which is insufficiently powered |
|  |  | Battin, 2001 | Small sample size which is insufficiently powered |
|  |  | Das, 2017 | Small sample size which is insufficiently powered |
|  |  | Gunn, 1998 | Mechanistic study |
|  |  | Sun, 2012 | Small sample size which is insufficiently powered |
| **Drug Intervention as Adjuvant** | **Magnesium** | Rahman, 2015 | Small sample size which is insufficiently powered |
|  |  | Gulczynska E, 2012 | Clinical biochemical responses reported. No outcome of interest. |
|  |  | Gulczynska E, 2014 | Clinical biochemical responses reported. No outcome of interest. |
|  | **Melatonin** | Aly, 2015 | Small sample size which is insufficiently powered |
|  | **Xenon** | Azzopardi, 2013 | Non-randomized. No control group. |
|  |  | Dingley J, 2014 | Cohort study. |
|  |  | Dingley J, 2015 | No outcome of interest. |
|  | **Erythropoietin** | Baserga, 2015 | Small sample size which is insufficiently powered |
|  |  | Rogers EE, 2014 | Non-randomized. No control group. All participants received intervention. |
|  |  | Valera IT, 2015 | All infants were in intervention group. |
|  |  | Wu YW, 2012 | Non-randomized. No control group. All participants received intervention. |
|  |  | Wu, 2016 | Small sample size which is insufficiently powered |
|  | **Topiramate** | Filippi L, 2012 | Study protocol. Study is still undergoing. |
|  |  | Filippi L, 2010 | Non-randomized observational study. |
|  |  | Filippi L, 2009 | All infants were given TPM. The study studied on pharmacokinetics. |
| . |  | Fillipi, 2017 | Poor outcome reporting with high dropout |

**eTable 3: Pairwise meta-analysis results for direct comparison of mortality endpoint and mortality or major neurodevelopmental disability assessed at least 18 months of age. All comparison were not significant unless bolded.**

| **Comparison** | **Number of studies** | **Odds ratio (95% CI)** | ***I*-squared (%)** | **Heterogeneity, χ^2^** |
| --- | --- | --- | --- | --- |
| **Mortality** | | | | |
| Erythropoietin vs usual care | 2 | 0.94 (0.39 -2.24) | 0 | 0.04 |
| Magnesium sulfate vs usual care | 2 | 0.80(0.21 – 3.11) | 0 | 0.03 |
| Selective head hypothermia vs usual care | 2 | 0.80 (0.54 – 1.19) | 0 | 0.29 |
| **Whole body hypothermia vs usual care** | 8 | 0.71 (0.54 – 0.92) | 0 | 0.04 |
| Whole body hypothermia with xenon vs whole body hypothermia | 1 | 1.22 (0.46 – 3.23) | - | - |
| **Mortality or major neurodevelopmental disability assessed at 18 months or later** | | | | |
| Erythropoietin vs usual care | 2 | 0.57 (0.36 – 0.91) | 0 | 0.00 |
| Selective head hypothermia vs usual care | 2 | 0.74 (0.53 – 1.04) | 0 | 0.56 |
| **Whole body hypothermia vs usual care** | 5 | 0.74 (0.59 – 0.92) | 0 | 2.73 |

**eTable 4**: Pairwise meta-analysis results for secondary outcomes examined in the studies. All comparison were not significant unless bolded.

| **Comparison** | **No of studies** | **Odds ratio (95% CI)** | ***I-*squared (%)** | **Heterogeneity, χ^2^** |
| --- | --- | --- | --- | --- |
| **Cerebral palsy** | | | | |
| Erythropoietin vs usual care | 2 | 0.47 (0.24 – 0.92) | 0 | 0.19 |
| **Whole body hypothermia vs usual care** | 7 | 0.70 (0.54 – 0.92) | 0 | 4.03 |
| Selective head hypothermia vs usual care | 2 | 0.80 (0.54 – 1.19) | 0 | 0.29 |
| Whole body hypothermia with xenon vs whole body hypothermia | 1 | 1.22 (0.46 – 3.23) | - | - |
| **Seizure** | | | | |
| Erythropoietin vs usual care | 1 | 0.47 (0.18 – 1.21) | - | - |
| Magnesium sulfate vs usual care | 1 | 0.93 (0.48 – 1.79) | - | - |
| Selective head hypothermia vs usual care | 2 | 0.80 (0.54 – 1.19) | 0 | 0.29 |
| **Whole body hypothermia vs usual care** | 5 | 0.73 (0.56 – 0.97) | 0 | 2.44 |
| Whole body hypothermia with xenon vs whole body hypothermia | 1 | 1.22 (0.46 – 3.23) | - | - |
| **Rate of severe neuromotor delay (Psychomotor developmental index score <70)** | | | | |
| Erythropoietin vs usual care | 1 | 0.60 (0.23 – 1.53) | - | - |
| Selective head hypothermia vs usual care | 1 | 0.74 (0.37 – 1.48) | - | - |
| Whole body hypothermia vs usual care | 3 | 0.82 (0.57 – 1.20) | 0 | 0.59 |
| **Rate of neurodevelopmental delay (Mental developmental index score <70)** | | | | |
| Erythropoietin vs usual care | 1 | 0.53 (0.20 – 1.40) | - | - |
| Selective head hypothermia vs usual care | 1 | 0.76 (0.39 – 1.50) | - | - |
| Whole body hypothermia vs usual care | 3 | 0.77 (0.53 – 1.12) | 0 | 0.17 |
| **Blindness** | | | | |
| Whole body hypothermia vs usual care | 6 | 0.60 (0.31 – 1.15) | 0 | 0.95 |
| Selective head hypothermia vs usual care | 2 | 0.44 (0.19 – 1.05) | 0 | 0.88 |
| Whole body hypothermia with topiramate vs whole body hypothermia | 1 | 1.00 (0.18 – 5.56) | - | - |
| **Hearing loss** | | | | |
| Erythropoietin vs usual care | 1 | 1.00 (0.14 – 7.43) | - | - |
| Whole body hypothermia vs usual care | 5 | 0.48 (0.22 – 1.06) | 0 | 1.55 |
| Selective head hypothermia vs usual care | 1 | 1.43 (0.33 – 6.27) | - | - |
| **Renal failure** | | | | |
| Erythropoietin vs usual care | 1 | 0.72 (0.36 – 1.43) | - | - |
| Whole body hypothermia vs usual care | 2 | 0.62 (0.31 – 1.22) | 0 | 0.03 |
| Selective head hypothermia vs usual care | 1 | 2.11 (0.38 –11.73) | - | - |
| **Sepsis** | | | | |
| Whole body hypothermia vs usual care | 4 | 0.91 (0.58 – 1.43) | 0 | 0.59 |
| Selective head hypothermia vs usual care | 1 | 2.11 (0.18 – 23.56) | - | - |
| Whole body hypothermia with xenon vs whole body hypothermia | 1 | 5.00 (0.23 – 107.01) | - | - |
| **Hypotension** | | | | |
| Erythropoietin vs usual care | 1 | 0.91 (0.48 – 1.71) | - | - |
| Whole body hypothermia vs usual care | 2 | 1.04 (0.81 – 1.34) | 0 | 1.08 |
| Selective head hypothermia vs usual care | 1 | 1.05 (0.26 - 4.32) | - | - |
| Whole body hypothermia with xenon vs whole body hypothermia | 1 | 1.07 (0.56 – 2.05) | - | - |
| **Hypoglycaemia** | | | | |
| Whole body hypothermia vs usual care | 5 | 0.89 (0.54 – 1.37) | 0 | 2.99 |
| **Bradycardia** | | | | |
| Whole body hypothermia vs usual care | 1 | 0.20 (0.01 – 4.26) | - | - |

**eTable 5**: Pairwise meta-analysis examining the effects of different therapies stratified by severity of encephalopathy. All comparison were not significant unless bolded.

| **Comparison** | **No of studies** | **Odds ratio (95% CI)** | ***I-*squared (%)** | **Heterogeneity, χ^2^** |
| --- | --- | --- | --- | --- |
| **Moderate encephalopathy** | | | | |
| **Erythropoietin vs usual care** | 2 | 0.27 (0.11 – 0.63) | 0 | 0.38 |
| **Whole body hypothermia vs usual care** | 5 | 0.63 (0.45 – 0.89) | 0 | 1.84 |
| Selective head hypothermia vs usual care | 2 | 0.68 (0.42 - 1.12) | 0 | 0.90 |
| **Severe encephalopathy** | | | | |
| Erythropoietin vs usual care | 2 | 0.74 (0.40 – 1.40) | 0 | 0.001 |
| Whole body hypothermia vs usual care | 5 | 0.87 (0.64 – 1.18) | 0 | 1.24 |
| Selective head hypothermia vs usual care | 2 | 0.76 (0.46 - 1.25) | 0 | 0.001 |

**eTable 6: Network meta-analysis for primary outcomes mortality and disability at 18 months. Comparisons should be read from left to right. The estimate is located at the intersection of the column-defining treatment and the row-defining treatment. An OR value below 1 favours the column-defining treatment. To obtain ORs for comparisons in the opposing direction, reciprocals should be taken. Any significant results are in bold and underlined.**

| **Whole body hypothermia** | 1.11 (0.59, 2.08) | 1.51 (0.68, 3.35) | **0.54 (0.32, 0.89)** |
| --- | --- | --- | --- |
|  | **Selective head hypothermia** | 1.37 (0.66, 2.81) | **0.49 (0.33, 0.72)** |
|  |  | **Erythropoietin** | **0.36 (0.29, 0.66)** |
|  |  |  | **Usual care** |

**eTable 7: Number needed to treat or number needed to harm for each intervention for the outcomes of mortality and mortality and neurodevelopmental delay at 18 months**

| **Comparison** | **NNTB (95% CI)** |
| --- | --- |
| **Mortality** |  |
| Whole body hypothermia vs usual care | 11 (7 – 26) |
| Selective head hypothermia vs usual care | 16 (NNTB 8 to ∞ to NNTH 44) |
| Erythropoietin vs usual care | 66 (NNTB 6 to ∞ to NNTH 1) |
| Magnesium sulfate vs usual care | 21 (NNTB 4 to ∞ to NNTH 4) |
| Xenon with whole body cooling vs usual care | 22 (NNTB 5 to ∞ to NNTH 5) |
| **Mortality and neurodevelopmental delay at 18 months** | |
| Whole body hypothermia vs usual care | 7 (4 – 34) |
| Selective head hypothermia vs usual care | 7 (4 – 27) |
| Erythropoietin vs usual care | 12 (3 – 16) |

Summaries showing the number needed to treat (benefit) (NNTB) value (95% confidence interval. NNTB to ∞ to number needed to harm (NNTH)) for each intervention.

**eTable 8: GRADE quality of evidence for the co-primary outcome of mortality or mortality and neurodevelopmental delay at 18 months. Using the GRADE criteria to rate the quality of evidence involved several steps namely: 1. Rating the quality of evidence for direct comparison; 2. Rating the quality of evidence for indirect estimates (starting at the lowest rating of the two pairwise direct estimates, which can be rated down further for transitivity or imprecision); 3. Rating the quality of evidence for network combining both direct and indirect estimates.**

| Comparison | Direct evidence | | Network meta-analysis | |
| --- | --- | --- | --- | --- |
|  | Odds ratio (95% CI) | Quality of evidence | Odds ratio (95% CI) | Quality of evidence |
| **Mortality** | | | | |
| EPO-UC | 0.94 (0.39 -2.24) | ⨁⨁◯◯^§†^  Low | 0.93 (0.39, 2.25) | ⨁⨁⨁◯  Moderate |
| MgS-UC | 0.80 (0.21 – 3.11) | ⨁⨁◯◯^§†^  Low | 0.79 (0.20, 3.06) | ⨁⨁◯◯  Low |
| WBH-UC | 0.71 (0.54 – 0.92) | ⨁⨁⨁⨁  High | 0.62 (0.46, 0.83) | ⨁⨁⨁⨁  High |
| SHH – UC | 0.80 (0.54 – 1.19) | ⨁⨁⨁◯^†^  Moderate | 0.73 (0.48, 1.11) | ⨁⨁⨁◯  Moderate |
| Xe+WBH - UC |  |  | 0.80 (0.28, 2.26) | ⨁◯◯◯ Very low |
| EPO-MgS |  |  | 1.17 (0.30, 4.55) | ⨁◯◯◯ Very low |
| WBH-MgS |  |  | 0.78 (0.20, 3.14) | ⨁◯◯◯ Very low |
| SHH – MgS |  |  | 0.92 (0.22, 3.83) | ⨁◯◯◯ Very low |
| Xe+WBH – MgS |  |  | 1.01 (0.18, 5.56) | ⨁◯◯◯ Very low |
| WBH-EPO |  |  | 0.66 (0.26, 1.67) | ⨁⨁◯◯  Low |
| SHH – EPO |  |  | 0.78 (0.30, 2.07) | ⨁◯◯◯ Very low |
| Xe+WBH – EPO |  |  | 0.85 (0.22, 3.33) | ⨁◯◯◯ Very low |
| SHH – WBH |  |  | 0.90 (0.48, 1.72) | ⨁⨁◯◯  Low |
| Xe+WBH – WBH | 1.22 (0.46 – 3.23) | ⨁⨁◯◯^§†^ Low | 1.29 (0.48, 3.49) | ⨁⨁◯◯  Low |
| Xe+WBH – SHH |  |  | 1.10 (0.36, 3.33) | ⨁◯◯◯ Very low |
| **Mortality or neurodevelopmental disability at 18 months** | | | | |
| EPO-UC | 0.57 (0.36 – 0.91) | ⨁⨁⨁◯^ǂ^  Moderate | 0.36 (0.29, 0.676 | ⨁⨁⨁◯  Moderate |
| WBH-UC | 0.74 (0.59 – 0.92) | ⨁⨁⨁⨁  High | 0.54 (0.32, 0.89) | ⨁⨁⨁⨁  High |
| SHH – UC | 0.74 (0.53 – 1.04) | ⨁⨁⨁◯^†^  Moderate | 0.49 (0.33, 0.72) | ⨁⨁⨁◯  Moderate |
| WBH – EPO |  |  | 1.51 (0.68, 3.35) | ⨁◯◯◯ Very low |
| SHH – EPO |  |  | 1.37 (0.66, 2.81) | ⨁◯◯◯ Very low |
| WBH – SHH |  |  | 1.11 (0.59, 2.08) | ⨁◯◯◯ Very low |

^§^Wide confidence intervals and few events; ^ǂ^ Contributing direct evidence of moderate quality with inadequate concealment of allocation and blinding; ^†^Only limited amount of studies contributing to direct evidence EPO- Erythropoietin; MgS- Magnesium sulfate; UC- Usual care; SHH- Selective head hypothermia; WBH- Whole body hypothermia; Xe- Xenon.

**eTable 9: Network meta-analysis results in sensitivity analysis. Pooled odds ratio of preventing mortality in sensitivity analyses based on (1) excluding studies conducted in low-middle income countries; and (2) restricting only studies in which had included term (>37 weeks) neonates. Numbers in parentheses indicate 95% credible interval.**

| **Comparison** | **Studies conducted in high income countries(1)** | **Full term neonates only (2)** |
| --- | --- | --- |
| Erythropoietin vs usual care | - | 0.93 (0.39 – 2.25) |
| Magnesium sulfate vs usual care | - | 0.79 (0.20 – 3.06) |
| Selective head cooling vs usual care | 0.81 (0.44 – 1.50) | 0.73 (0.48 – 1.11) |
| Whole body cooling vs usual care | **0.64 (0.46 – 0.90)** | **0.50 (0.31 – 0.81)** |
| Whole body cooling with xenon vs usual care | 0.83 (0.28 – 2.45) | - |
| **Mortality or major neurodevelopmental disability assessed at 18 months or later** | | |
| Erythropoietin vs usual care | - | 0.36 (0.21 – 0.61) |
| Selective head cooling vs usual care | 0.61 (0.28 – 1.33) | **0.54 (0.36 – 0.81)** |
| Whole body cooling vs usual care | **0.51 (0.33 – 0.78)** | **0.44 (0.27 – 0.71)** |

Outcome (1) – 4 trials whole body cooling vs usual care; 1 trials selective head cooling vs usual care; and 1 trial xenon with whole body cooling vs whole body cooling

Outcome (2) – 2 trials erythropoietin vs usual care; 2 trial magnesium sulfate vs usual care; 5 trials whole body cooling vs usual care; and 2 trials selective head cooling vs usual care

**Risk of bias assessment**

Risk of bias assessment followed the recommended approach for assessing risk of bias by Cochrane. Specific bias domains addressed include methods for random sequence generation, allocation concealment, blinding of participants and investigators, blinding of outcome assessment, incompleteness of outcome data as well as selective outcome reporting. The adjudication of the risk of bias for each item was performed by answering pre-specified questions regarding the methodology reported by each study. The conclusion on risk of bias was then classified as either low, unclear or high risk of bias. Most studies had a high risk of bias for blinding of participants and personnel since blinding could not be performed especially in studies which examined the use of hypothermia.

eFigure 1: Quality assessment of included studies for all studies included in the review, using the Cochrane’s risk of bias assessment tool presented as percentages (15 studies).


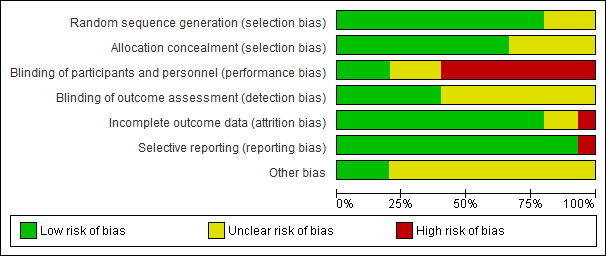


**eFigure 2: Network plots for outcomes (a) mortality, (b) mortality or disability at 18 months, (c) seizures, (d) cerebral palsy, (e) rate of severe neuromotor delay, (f) rate of severe neuoromotor developmental delay, (g) blindness, (h) hearing loss, (i) renal failure, (j) sepsis, and (k) hypotension for infants with hypoxic ischemic encephalopathy. The presence of a line between nodes indicates that the comparison has been compared directly within a trial. The size of nodes and thickness of the edges are weighted according to the number of studies evaluating each treatment and direct comparison, respectively. EPO- Erythropoietin; Magnesium- Magnesium sulfate; Head hypothermia - Selective head hypothermia; Body hypothermia- Whole body hypothermia; Xe- Xenon**

1. Mortality


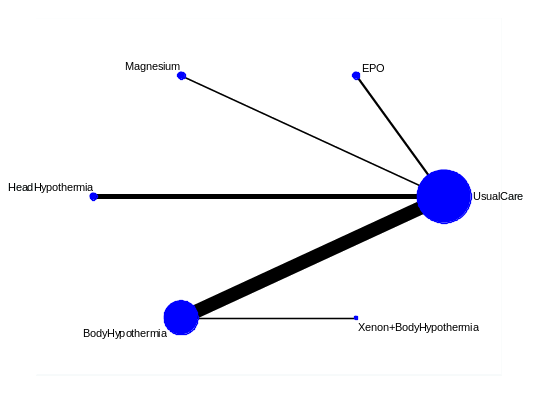


1. Mortality or disability at 18 months


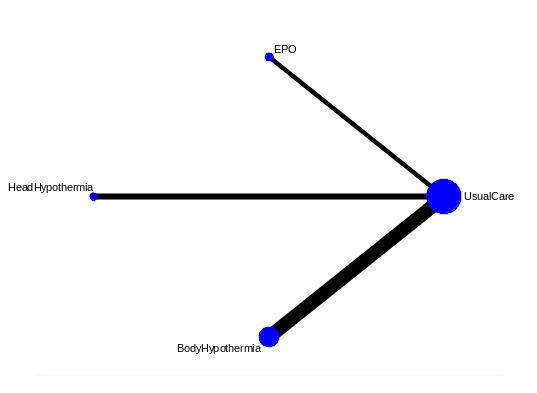


1. Seizures


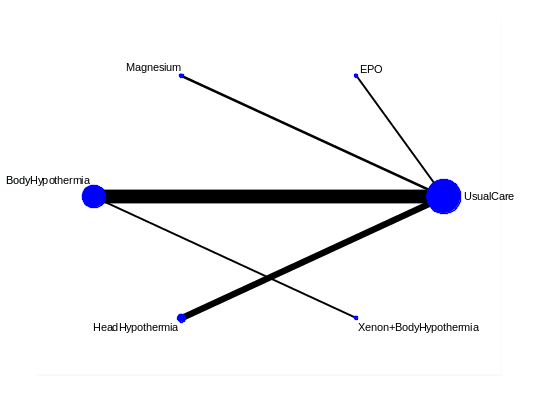


1. Cerebral palsy
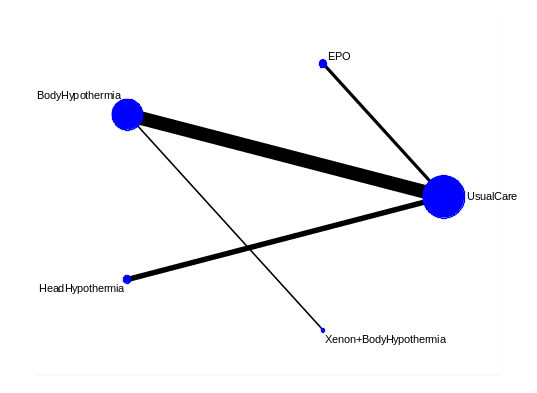

2. Rate of severe neuromotor delay (PDI score <70)


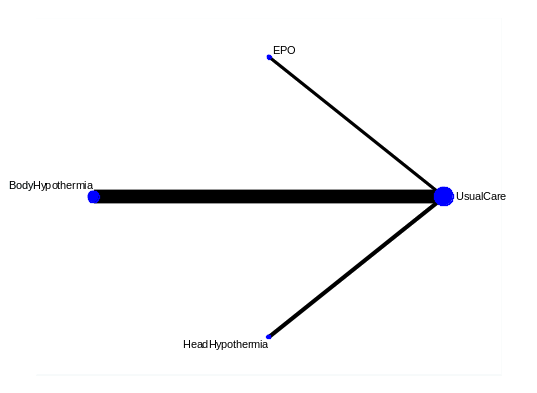


1. Rate of severe neurodevelopmental delay (MDI score <70)


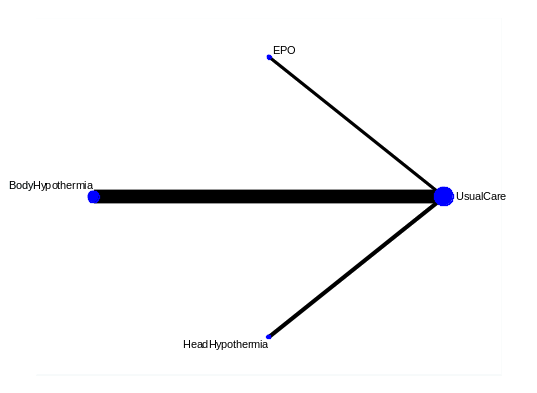


1. Blindness


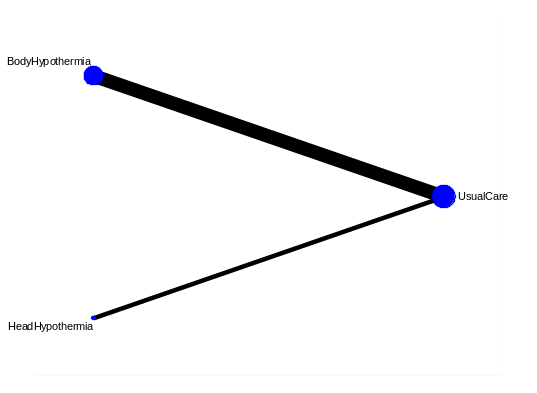


1. Hearing loss


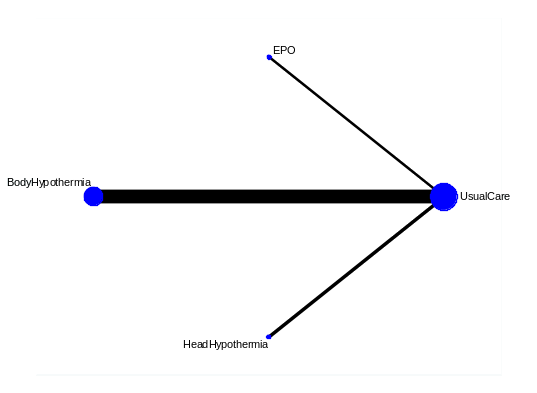


1. Renal failure


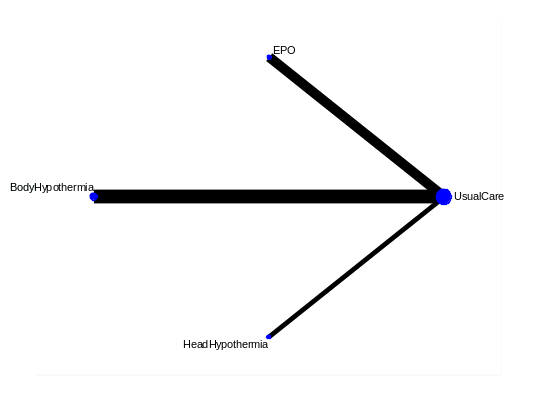


1. Sepsis


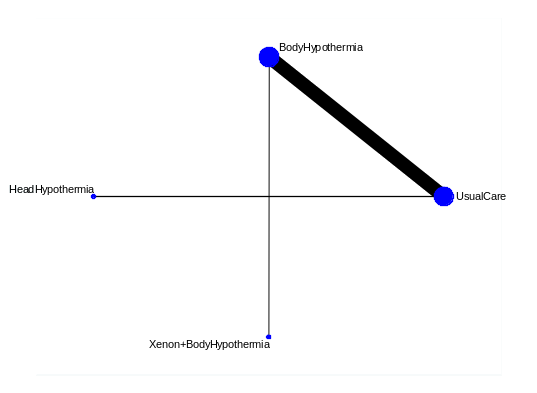


1. Hypotension


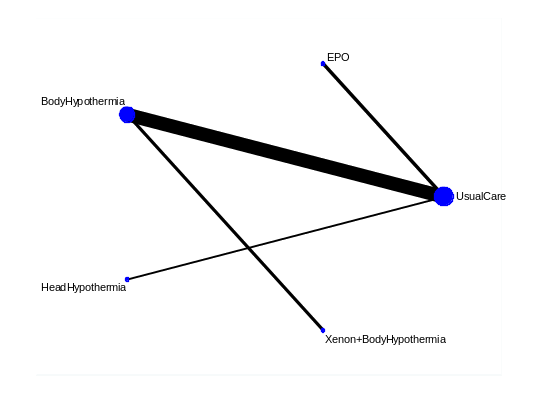


**eFigure 3:Network meta-analysis forest plots of different treatments compared to standard care on mortality or neurodevelopmental disability stratified by severity of encephalopathy. EPO- Erythropoietin; SHC - Selective head hypothermia; WBC- Whole body hypothermia**


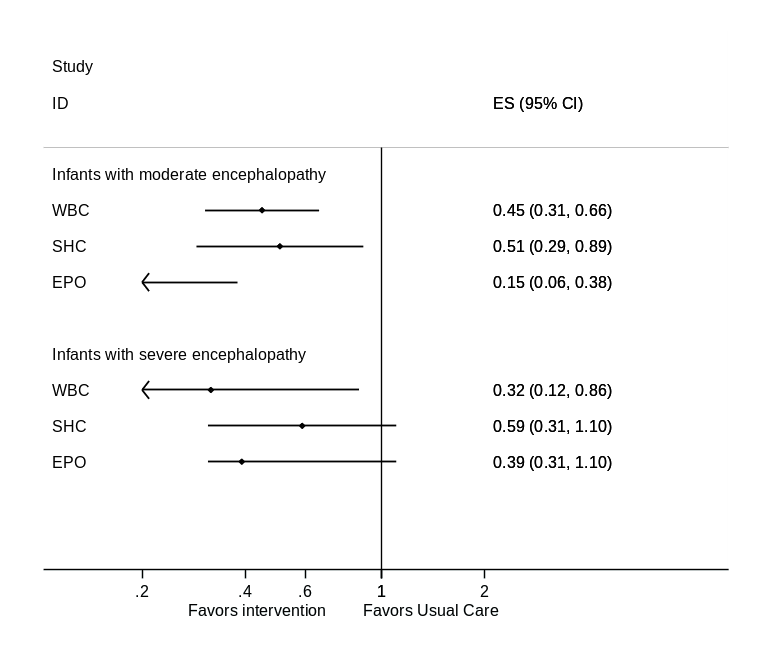


**Intervention Odds ratio (95% Credible Interval)**

**eFigure 4: Comparison adjusted funnel plots for outcomes assessed**

1. Mortality


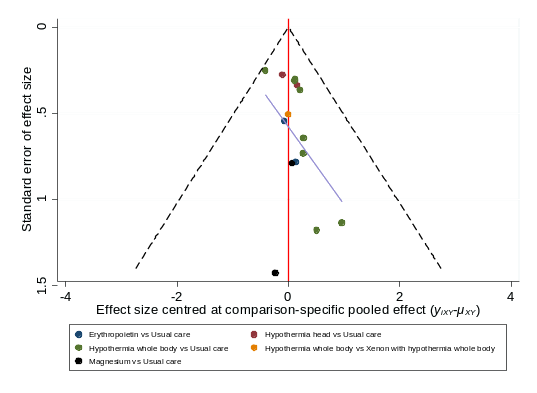


1. Mortality or neurodevelopmental delay at 18 months


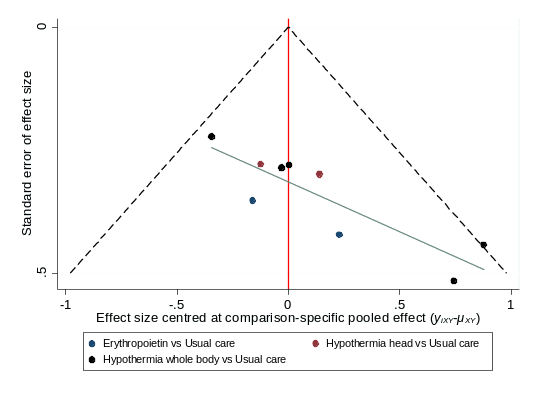


1. Cerebral palsy


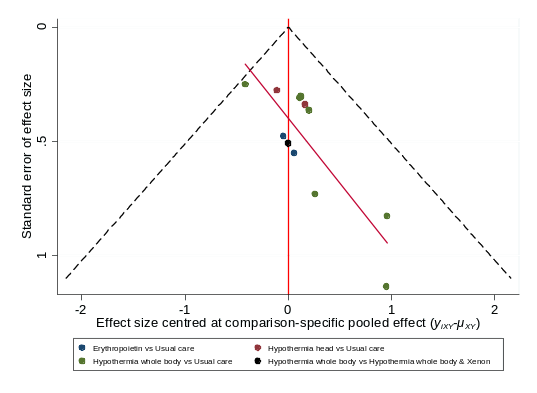


1. Seizures


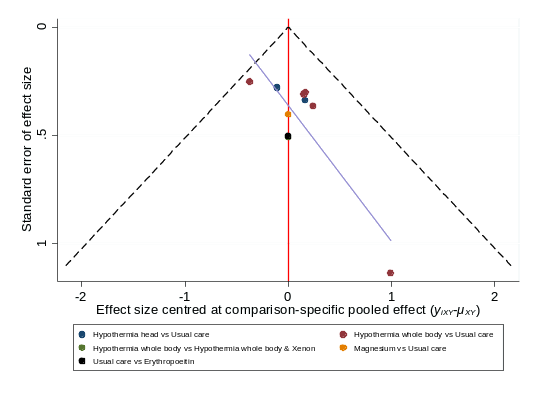


1. Rate of severe neuromotor delay (PDI score <70)


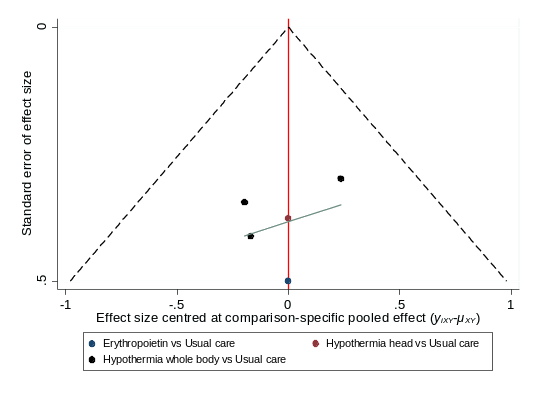


1. Rate of severe neurodevelopmental delay (MDI score <70)


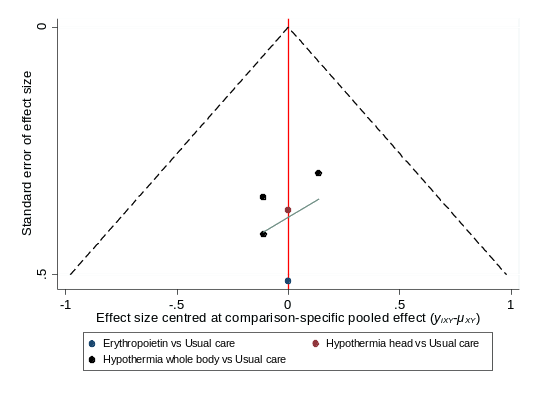


1. Blindness


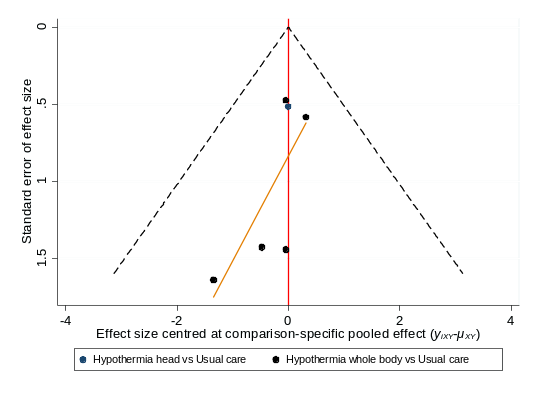


1. Hearing loss


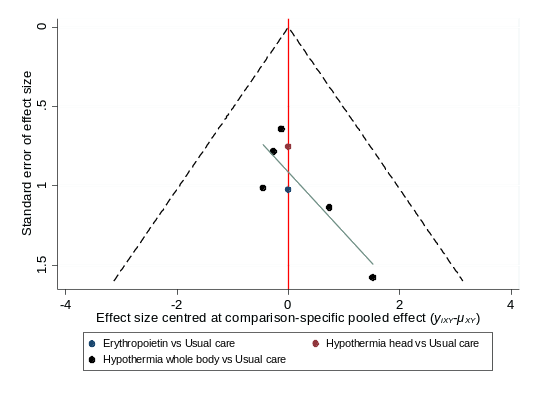


1. Renal failure
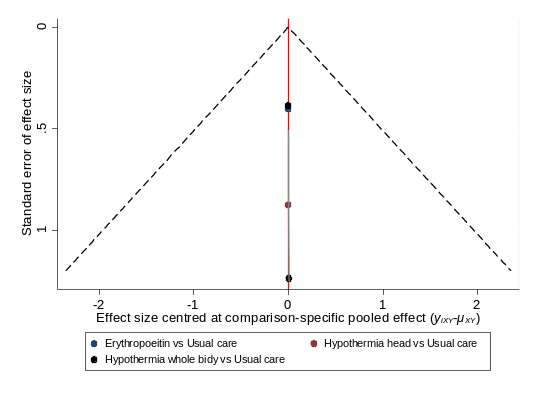

2. Sepsis


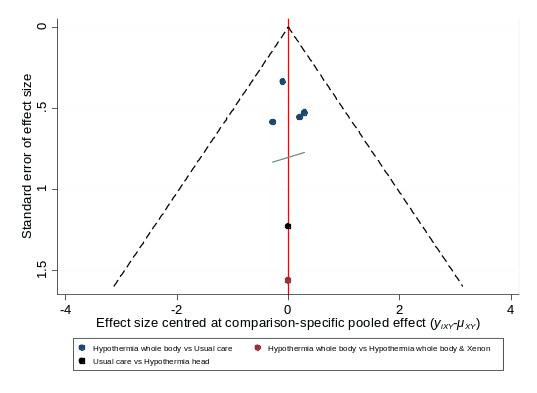


1. Hypotension


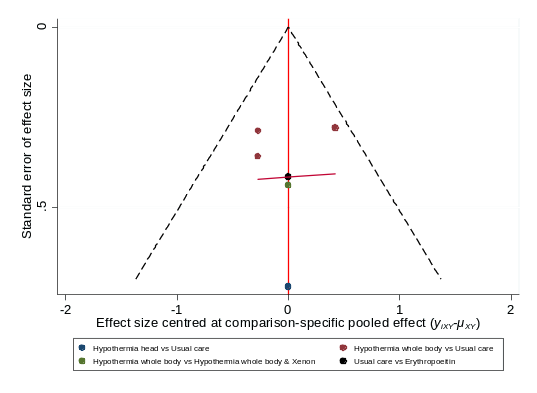


**eFigure 5: Ranking of treatment based upon SUCRA values for outcomes assessed. EPO- Erythropoietin; Magnesium- Magnesium sulfate; Usual - Usual care; CoolingHead- Selective head hypothermia; CoolingBody- Whole body hypothermia; Xe- Xenon**

1. Mortality

Treatment ranking

| **Treatment** | **SUCRA (%)** |
| --- | --- |
| Usual care | 24.5 |
| Erythropoietin | 37.5 |
| Magnesium sulfate | 50.3 |
| Whole body hypothermia | 77.8 |
| Selective head hypothermia | 60.2 |
| Whole body hypothermia with xenon | 49.7 |

*Larger SUCRA represents more effective intervention


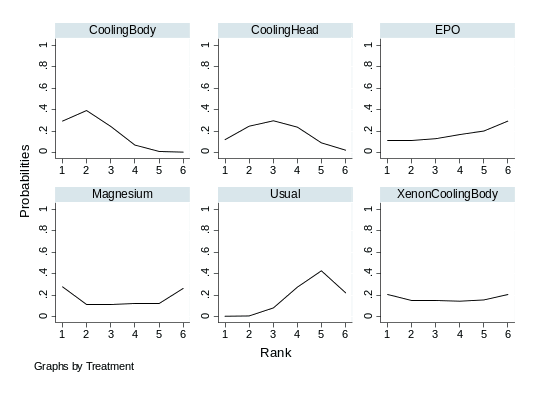


1. Morbidity or mortality at 18 months

Treatment ranking

| **Treatment** | **SUCRA (%)** |
| --- | --- |
| Usual care | 0.2 |
| Erythropoietin | 88.8 |
| Whole body hypothermia | 50.6 |
| Selective head hypothermia | 60.4 |

*Larger SUCRA represents more effective intervention


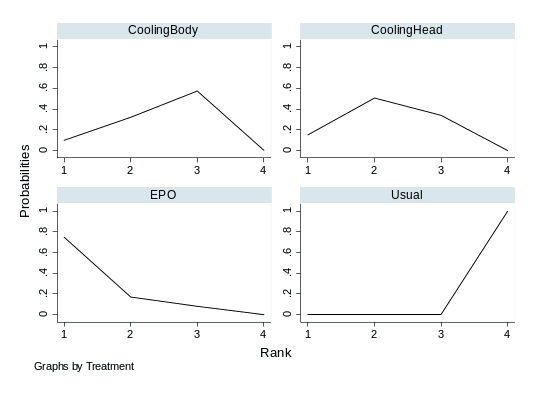


1. Cerebral palsy

**Treatment ranking**

| **Treatment** | **SUCRA (%)** |
| --- | --- |
| Usual care | 10.6 |
| Erythropoietin | 93.1 |
| Whole body hypothermia | 63.6 |
| Selective head hypothermia | 44.8 |
| Whole body hypothermia with xenon | 38.0 |

*Larger SUCRA represents more effective intervention


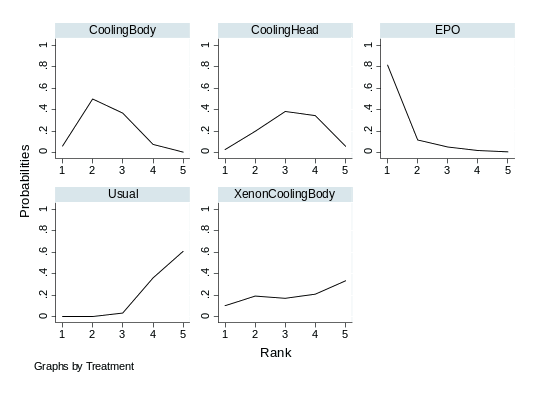


(d) Seizures outcome

Treatment ranking

| **Treatment** | **SUCRA (%)** |
| --- | --- |
| Usual care | 16.0 |
| Erythropoietin | 90.9 |
| Magnesium sulfate | 37.1 |
| Whole body hypothermia | 64.9 |
| Selective head hypothermia | 50.6 |
| Whole body hypothermia with xenon | 40.5 |

*Larger SUCRA represents more effective intervention


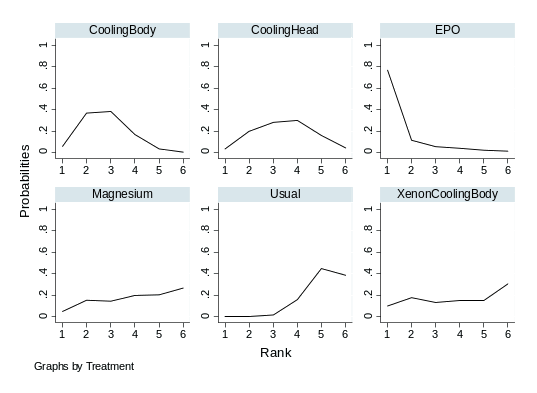


(e) Rate of severe neuromotor delay (PDI score <70)

Treatment ranking

| **Treatment** | **SUCRA (%)** |
| --- | --- |
| Usual care | 9.1 |
| Erythropoietin | 79.9 |
| Whole body hypothermia | 48.1 |
| Selective head hypothermia | 62.8 |

*Larger SUCRA represents more effective intervention


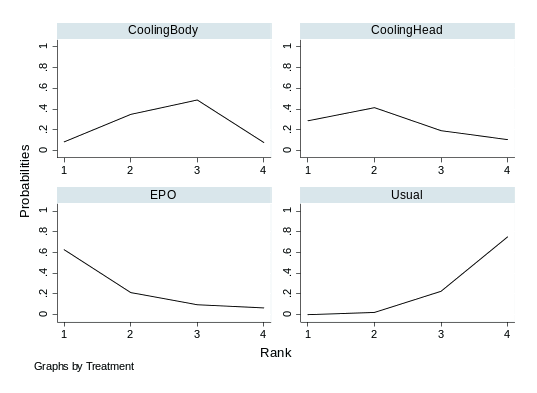


(f) Rate of severe neurodevelopmental delay (MDI score <70)

Treatment ranking

| **Treatment** | **SUCRA (%)** |
| --- | --- |
| Usual care | 6.9 |
| Erythropoietin | 85.2 |
| Whole body hypothermia | 51.8 |
| Selective head hypothermia | 56.1 |

*Larger SUCRA represents more effective intervention


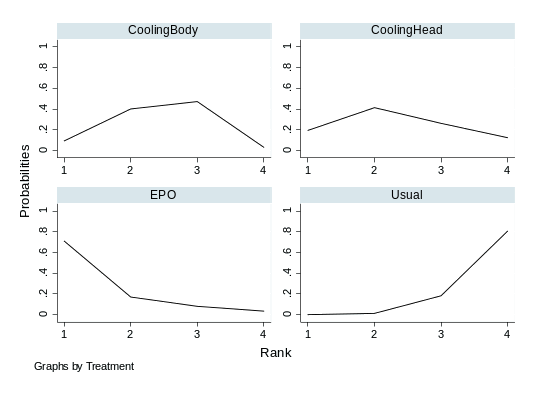


(g) Hearing loss

Treatment ranking

| **Treatment** | **SUCRA (%)** |
| --- | --- |
| Usual care | 40.3 |
| Erythropoietin | 45.6 |
| Whole body hypothermia | 87.8 |
| Selective head hypothermia | 26.3 |

*Larger SUCRA represents more effective intervention


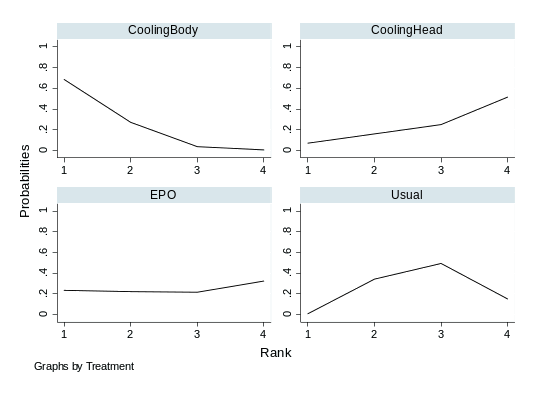


(h) Blindness

Treatment ranking

| **Treatment** | **SUCRA (%)** |
| --- | --- |
| Usual care | 8.2 |
| Whole body hypothermia | 69.0 |
| Selective head hypothermia | 72.9 |

*Larger SUCRA represents more effective intervention


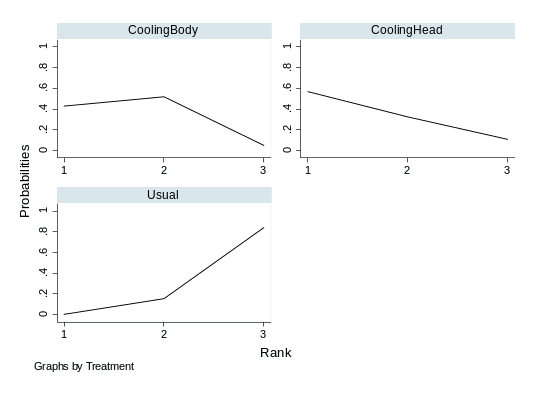


(i) Renal failure

Treatment ranking

| **Treatment** | **SUCRA (%)** |
| --- | --- |
| Usual care | 29.6 |
| Erythropoietin | 77.9 |
| Whole body hypothermia | 81.2 |
| Selective head hypothermia | 11.3 |

*Larger SUCRA represents more effective intervention


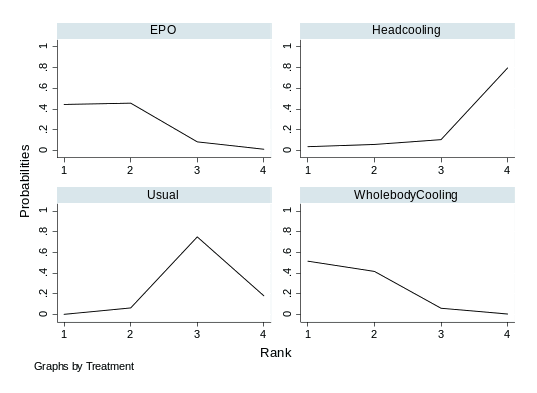


(j) Sepsis

Treatment ranking

| **Treatment** | **SUCRA (%)** |
| --- | --- |
| Usual care | 63.3 |
| Whole body hypothermia | 76.0 |
| Selective head hypothermia | 38.7 |
| Whole body hypothermia with xenon | 21.9 |

*Larger SUCRA represents more effective intervention


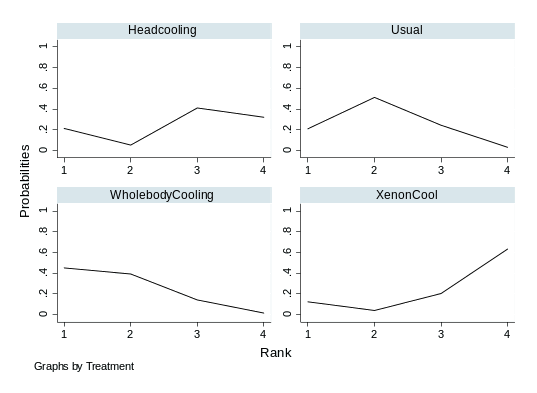


(k) Hypotension

Treatment ranking

| **Treatment** | **SUCRA (%)** |
| --- | --- |
| Usual care | 54.6 |
| Erythropoietin | 71.0 |
| Whole body hypothermia | 43.1 |
| Selective head hypothermia | 48.6 |
| Whole body hypothermia with xenon | 32.7 |

*Larger SUCRA represents more effective intervention


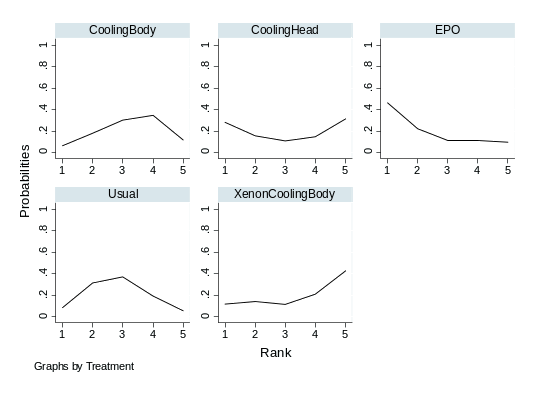

Supplement: Supplementary file 1 [file Table_1.doc]
